# Aberrant Methylation of Aging-Related Genes in Asthma

**DOI:** 10.3389/fmolb.2021.655285

**Published:** 2021-05-25

**Authors:** Yu Yang, Lin Yuan, Ming Yang, Xizi Du, Ling Qin, Leyuan Wang, Kai Zhou, Mengping Wu, Ruoxi He, Juntao Feng, Yang Xiang, Xiangping Qu, Huijun Liu, Xiaoqun Qin, Chi Liu

**Affiliations:** ^1^ Department of Respiratory Medicine, National Clinical Research Center for Respiratory Diseases, Xiangya Hospital, Central South University, Changsha, China; ^2^ Department of Physiology, School of Basic Medicine Science, Central South University, Changsha, China; ^3^ Basic and Clinical Research Laboratory of Major Respiratory Diseases, Central South University, Changsha, China; ^4^ Centre for Asthma and Respiratory Disease, School of Biomedical Sciences and Pharmacy, Faculty of Health and Medicine, University of Newcastle and Hunter Medical Research Institute, Callaghan, NSW, Australia; ^5^ Research Center of China-Africa Infectious Diseases, Xiangya School of Medicine Central South University, Changsha, China

**Keywords:** aging-related genes, DNA methylation, aging, asthma, allergy

## Abstract

**Background:** Asthma is a complex pulmonary inflammatory disease which is common among older adults. Aging-related alterations have also been found in structural cells and immune cells of asthma patients. Nonetheless, the underlying mechanism by which differenced aging-related gene contributes to asthma pathology remains unclear. Of note, DNA methylation (DNAm) has been proven to play a critical mechanism for age-related gene expression changes. However, the methylation changes of aging-related genes in asthma patients are still obscure.

**Methods:** First, changes in DNAm and gene expression were detected with multiple targeted bisulfite enrichment sequencing (MethTarget) and qPCR in peripheral blood of 51 healthy controls (HCs) and 55 asthmatic patients. Second, the correlation between the DNAm levels of specific altered CpG sites and the pulmonary function indicators of asthma patients was evaluated. Last, the receiver operator characteristic (ROC) curve and principal component analysis (PCA) were used to identify the feasibility of the candidate CpG sites as biomarkers for asthma.

**Results:** Compared with HCs, there was a differential mRNA expression for nine aging-related genes in peripheral blood of asthma patients. Besides, the methylation levels of the nine aging-related genes were also altered in asthma patients, and a total of 68 CpG sites were associated with the severity of asthma. Notably, 9 of the 68 CpG sites were significantly associated with pulmonary function parameters. Moreover, ROC curve and PCA analysis showed that the candidate differential methylation sites (DMSs) can be used as potential biomarkers for asthma.

**Conclusions:** In summary, this study confirmed the differentially expressed mRNA and aberrant DNAm level of aging-related genes in asthma patients. DMSs are associated with the clinical evaluation indicators of asthma, which indicate the involvement of aging-related genes in the pathogenesis of asthma and provide some new possible biomarkers for asthma.

## Introduction

Asthma is a chronic and complex pulmonary inflammation disease which is characterized by aberrant immune responses to allergen, reversible airflow obstruction, and airway hyperresponsiveness (AHR). Although bronchodilators and inhaled/systemic corticosteroids are highly effective in most asthma patients, approximately 5–10% asthma patients are still steroid-refractory, which always have lower lung function and higher mortality (Luhadia, 2014; Maltby et al., 2017). Classical “allergic constitution” or “airway inflammation” cannot fully explain the occurrence and development of asthma. Thus, accumulating studies are attempted to further elucidate the inner pathogenesis of asthma and identify novel therapeutic targets.

Intriguingly, asthma is common among older adults (aged over 65 years), which is usually more severe, with little opportunities of remission (Dunn et al., 2018). Accumulative studies have demonstrated the involvement of aging in the parthenogenesis of chronic pulmonary diseases, including idiopathic pulmonary fibrosis (IPF) and chronic obstructive pulmonary diseases (COPD). As is known, the pathological changes in asthma resemble those in COPD, such as airway remodeling, chronic inflammation, and decreased lung function (Zhou-Suckow et al., 2017; Aghasafari et al., 2019). It is feasible to speculate the possible involvement of aging in the development of asthma. Indeed, some valuable evidences have implicated that aging is a vital dangerous factor for the development of asthma (Budde and Skloot, 2018). Aging-related changes have also been found in structural cells and immune cells of asthma patients. Of particular note is that the hallmarks of aging such as telomere attrition, epigenetic alterations, loss of proteostasis, and altered intercellular communication have also been detected in asthma patients (Kennedy et al., 2014). Besides, aging can affect asthma severity along with its diagnosis and management, which is significant for the treatment of asthma (Budde and Skloot, 2018). The aging of different targeted cells can also contribute to the pathobiology of asthma, including airway inflammation, airway remodeling, and decreased lung function (Wang et al., 2020). Furthermore, it has been confirmed that antiaging strategies can improve pathological processes such as airway inflammation and airway remodeling in asthma patients (Conte et al., 2015).

Although more and more undeniable studies have evidenced the association between aging and asthma, the role of aging and the mechanism behind the differential expression of aging-related genes are still obscure. A series of recent researches have confirmed that epigenetic mechanisms are involved in the regulation of the expression of aging-related genes (Johnson et al., 2012; Field et al., 2018). Epigenetic mechanisms containing DNA methylation (DNAm), microRNA expression, and histone modifications could regulate the transcription activities of the target genes without alteration of the nucleotide sequence. In particular, DNAm is the most deeply studied epigenetic regulation, which has been proven to play a crucial role in the regulation of aging-related genes (Yang et al., 2014). Specifically, it has been verified that cytosine methylation at the CpG site affected multiple regulatory mechanisms of aging-related genes during transcription (Zhu et al., 2016; Morales-Nebreda et al., 2019) and further participated in aging-related diseases such as asthma and COPD (Nicodemus-Johnson et al., 2016; Morrow et al., 2016; Morrow et al., 2018). A series of previous studies have verified that DNAm regulations are involved in the pathogenesis of respiratory diseases such as allergies and asthma (DeVries and Vercelli, 2016; Miller and Lawrence, 2018; Peng et al., 2019). However, the DNAm mutations of aging-related genes in asthma patients are still obscure.

Our previous study screened and evaluated the differential mRNA expression and altered methylation levels of nine aging-related genes (AREG, ATG3, E2F1, FOXO3, HDAC1, MMP2, NUF2, TGFB1, and TP53) in COPD patients (Du et al., 2019). It is found that DNAm was involved in regulating the expression of nine aging-related genes in peripheral venous blood of COPD patients. Besides, the methylation level of certain special CpG sites was associated with the incidence and severity of COPD (Du et al., 2019). In this study, we further aim to probe the potential involvement of these previously screened nine aging-related genes in the parthenogenesis of asthma. First, we inspected the changes in DNAm and mRNA expression of the nine aging-related genes in peripheral venous blood of healthy controls (HCs) and asthmatic patients. Then, we analyzed the correlation between DMSs and clinical indicators in asthmatic patients. Finally, we assessed the feasibility of the candidate CpG sites as biomarkers for asthma.

## Methods

### Subjects and Data Collection

The study was approved by no. 20180308 of the Xiangya Hospital Ethics Review Committee. From October 2018 to January 2019, 51 HCs and 55 asthma patients were chosen from the Respiratory Department and Physical Examination Center of Xiangya Hospital, China. FEV_1_/FVC ratio <0.7 and FEV_1_% <70% were defined as the presence of asthma. The inclusive standards for the patient group were between the age of 40 and 70 years with a clear diagnosis of asthma (according to the criteria of 2018 Global Strategy for Asthma Management and Prevention) but without other respiratory or other diseases (GINA Report, 2021). The HCs had no differences in age and gender without asthma or other organic mental diseases, including smoking and nonsmoking controls. Quality control methods were strictly enforced.

After obtaining the written informed consent from each subject, we collected questionnaire information (general condition, smoking history, and other respiratory diseases), pulmonary function testing, and peripheral blood samples. For our analysis, pulmonary function parameters including forced expiratory volume in 1 s as percentage of predicted volume (FEV_1_%), the spirometric values of forced expiratory volume in 1 s (FEV_1_), forced vital capacity (FVC), peak expiratory force (PEF), and forced expiratory flow (FEF) were adopted. Certified staff performed all interviews and examinations. Moreover, feedback on work quality would be regularly provided to field staff during the data collection process, and secondary training would be conducted when necessary.

### Sample Collection

A total of 106 whole blood samples were collected from the enrolled 51 HCs and 55 asthma patients. Then, the collected peripheral blood was placed into 5 ml EDTA anticoagulation tubes and transferred to a centrifuge tube. After adding 2 volumes of erythrocyte lysate and lysing for 5 min, peripheral blood cells were pelleted by centrifugation and stored at −80°C.

### RNA Extraction and Quantitative RT-PCR

Total mRNA was purified from peripheral blood cells using Trizol (Invitrogen) and quantified by an ultraviolet spectrophotometer (Thermo Fisher Scientific, MA, United States) (Yuan et al., 2019). 1 μg RNA was reverse-transcribed into cDNA using Reverse Transcriptase Kit (Qiagen, Netherlands) in accordance to the manufacturer’s instructions (Yuan et al., 2020). Then, quantitative RT-PCR was executed using SYBR^®^ Premix Ex Taq™ II system (TaKaRa, Japan) with the CFX96 Touch™ Real-Time PCR Detection System (Bio-Rad, CA, United States). 1 μL of the reverse-transcript was added to a 30-μL PCR mixture for 40 cycles. Each cycle included 93°C for 30 s and 54°C for 60 s. By the comparison between the copy numbers of target gene and *β*-actin, the normalization of mRNA expression data for sample-to-sample variability in RNA input, RNA quality, and reverse transcription efficiency was completed. Primer sequences are described in Table 1.

**TABLE 1:** Primer sequence of aging-related genes for qPCR.

Gene	Primer	
AREG	Forward	TGT​CGC​TCT​TGA​TAC​TCG​GC
	Reverse	AGG​CAT​TTC​ACT​CAC​AGG​GG
ATG3	Forward	GTG​TTC​AGT​TCA​CCC​ATG​CAG
	Reverse	TTA​ACA​GCC​ATT​TTG​CCA​CTA​ATC​T
E2F1	Forward	CAT​CCC​AGG​AGG​TCA​CTT​CTG
	Reverse	GAC​AAC​AGC​GGT​TCT​TGC​TC
FOXO3	Forward	CGG​ACA​AAC​GGC​TCA​CTC​T
	Reverse	GGA​CCC​GCA​TGA​ATC​GAC​TAT
HDAC1	Forward	TTTTTGGGTYGGAYGTTGAG
	Reverse	CCCTCRCAACCTCCTCTCC
MMP2	Forward	TGG​CAC​CCA​TTT​ACA​CCT​AC
	Reverse	CCT​CGT​ATA​CCG​CAT​CAA​TC
NUF2	Forward	TGT​TAA​GCA​ATA​CAA​ACG​CAC​AG
	Reverse	TGC​CTT​TTC​AAT​ACC​GTC​GTG
TGFB1	Forward	CGA​CTC​GCC​AGA​GTG​GTT​AT
	Reverse	GCT​AAG​GCG​AAA​GCC​CTC​AA
TP53	Forward	AAG​TCT​GTG​ACT​TGC​ACG​TAC​TCC
	Reverse	GTC​ATG​TGC​TGT​GAC​TGC​TTG​TAG
β-actin	Forward	TTC​CAG​CCT​TCC​TTC​CTG​GG
	Reverse	TTG​CGC​TCA​GGA​GGA​GCA​AT

### DNA Extraction, Bisulfite Treatment, and Methylation Array Methods

A commercially available kit (TIANGEN Biotech, Beijing, China) was used to extract genomic DNA from whole blood according to previous publications (Koshy et al., 2017). Genesky Biotechnologies Inc. performed bisulfite processing, methylation library construction, high-throughput sequencing, and quality control (Li et al., 2017). CpG islands located between 2 K upstream of the gene transcription start site and 1 K downstream of the first exon were selected to measure the methylation level. 18 CpG islands from the nine screened aging-related genes were selected (two from AREG, two from ATG3, one from E2F1, three from FOXO3, one from HDAC1, three from MMP2, one from NUF2, three from TGFB1, and two from TP53) according to our previous publications (Du et al., 2019). Then, bisulfite modification of DNA sample, methylation library construction, and MethTarget were performed (Du et al., 2019). 856 CpG sites from nine distinguishingly expressed aging-related genes in the methylation assay were detected. We only selected the original data with a sequencing quality value of Q > 40 (basic sequencing error rate <0.1%), and the methylation percentage of each CpG site was presented. In the process of sequencing, due to the sample getting segmented into multiple fragments during amplification, a few fragments were detected repeatedly, which was specifically labeled in the results.

### Statistical Analysis

The characteristic data of all recruited HCs and asthma patients were shown as mean ± SD, *p*-value < 0.05, and analyzed by unpaired *t* test. *t* test and nonparametric test (Mann–Whitney *U* test) were used to analyze the mRNA expression and the methylation array of AREG, ATG3, E2F1, FOXO3, HDAC1, MMP2, NUF2, TGFB1, and TP53. We used the Benjamini Hochberg method to control the false discovery rate (FDR). The selection of distinguishingly expressed CpG sites was performed by logistic regression analysis, with latent risk factors of age and gender (Miravitlles et al., 2000). The correlation between the percentage of methylation of candidate CpG sites and successive variables for instance FEV_1_%, FVC, FEV_1_, and PEF was assessed by Pearson’s correlation or Spearman’s correlation. ROC analysis was obtained to elucidate the accuracy of candidate DMSs or methylation change rates in predicting asthma. For each candidate DMS, the optimal cutoff value for predicting asthma and corresponding sensitivity and specificity were defined by the maximum Youden index value (sensitivity + specificity-1) (Fluss et al., 2005). The methylation percentage of candidate DMSs or the methylation status (change or not change) were used for PCA to identify asthma. For each candidate DMS, the change in methylation status was defined by its optimal threshold (Saito et al., 2017). The methylation change rate in each sample mainly referred to the probability that the methylation status of the candidate DMSs changed. The statistical analyses were implemented using SPSS version 22.0 (IBM Corporation, Armonk, NY, United States). A two-tailed *p*-value <0.05 was considered statistically significant, ****p < 0.0001; *p < 0.05.

## Results

### Differential Expression of the Nine Screened Aging-Related Genes in Peripheral Blood of Asthma Patients

**FIGURE 1:**
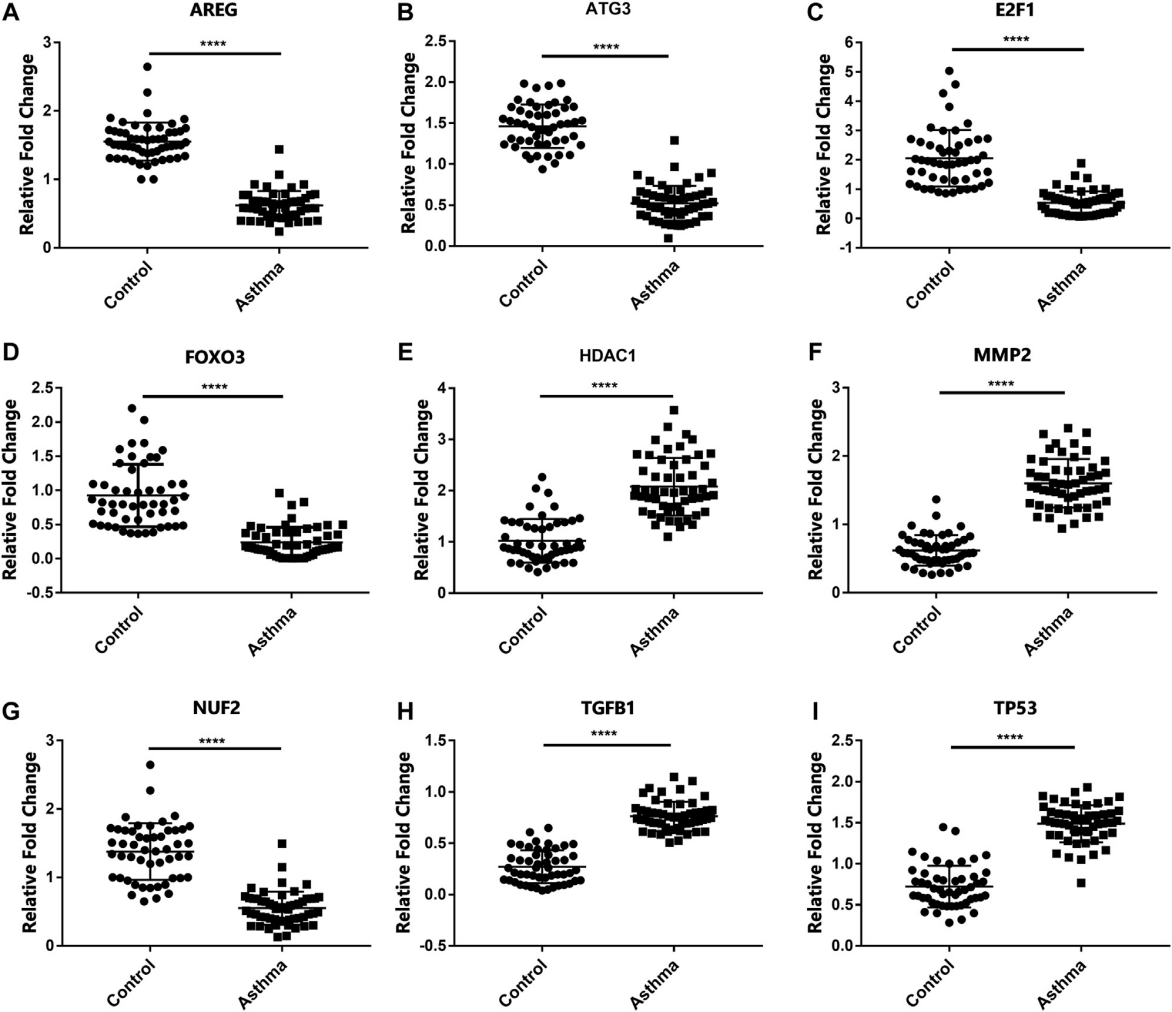
mRNA levels of aging-related genes in HCs and asthma patients. **(A–I)** The mRNA expression of AREG, ATG3, E2F1, FOXO3, HDAC1, MMP2, NUF2, TGFB1, and TP53 in HCs and asthma patients. ****p < 0.0001.

In order to detect the expression of the previously screened nine aging-related genes in asthma patients, peripheral blood was collected from 51 HCs and 55 asthma patients. The demographic characteristics of all the subjects are shown in Table 2. There was no significant difference in age between asthma patients and HCs. Compared with HCs, the mRNA expression of AREG, ATG3, E2F1, FOXO3, HDAC1, MMP2, NUF2, TGFB1, and TP53 in the asthma group changed significantly (Figure 1).

**TABLE 2:** Demographic characteristics of asthma patients and HCs.

	Control	Asthma
Number of subjects	51	55
Age	53.83 ± 6.84	46.72 ± 10.41
Gender (f/m)	41/10	46/9
FEV_1_	2.82 ± 0.20	1.76 ± 0.62^*^
FEV_1_% predicted	0.92 ± 0.25	0.70 ± 0.24^*^
FVC	4.02 ± 0.65	2.84 ± 0.85^*^
FEV_1_/FVC	0.83 ± 0.03	0.65 ± 0.16^*^
PEF	8.34 ± 0.92	4.65 ± 0.85^*^
FEF_75_	0.86 ± 0.35	0.47 ± 0.22^*^
FEF_50_	0.83 ± 0.34	0.37 ± 0.16^*^
FEF_25_	0.72 ± 0.22	0.23 ± 0.18^*^

Data are presented as mean ± SD.

* p-value < 0.05, asthma patients vs controls (unpaired t test).

### Altered Methylation Levels of the Nine Aging-Related Genes in Peripheral Blood of Asthma Patients

**FIGURE 2:**
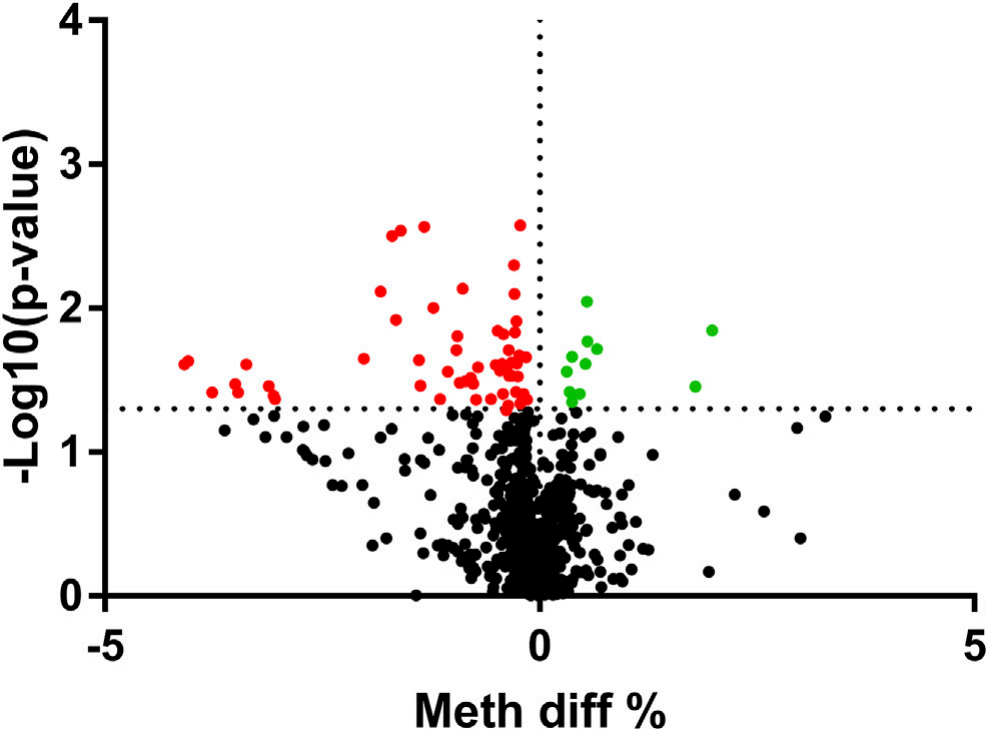
Volcano plot of the differential methylation CpG sites between HCs and asthma patients. The upregulated sites were presented as green dots, and downregulated sites were presented as red dots. *p＜0.05 sites were presented above the dotted line.

As the mRNA expression of the nine aging-related genes altered significantly in asthma patients, we further determined the methylation levels of the nine aging-related genes in asthma patients. We analyzed the total 856 CpG sites in the CpG islands of the nine aging-related genes. The methylation analysis result was shown *via* volcano maps (Figure 2). It is shown that the methylation levels of 68 CpG sites were related to asthma at FDR <5%. The detailed information of all the differential 68 DMSs is demonstrated in Supplementary Table S1. In addition, we analyzed the correlation between the methylation level of the 68 CpG sites and the expression of the corresponding aging-related genes. Among all the 68 CpG sites, there is a negative association between mRNA expression and DNAm in 58 CpG sites (Supplementary Table S2). This correlation strongly indicates that the methylation level of the CpG sites would have a negative impact on the expression of the corresponding aging-related genes.

### Potential Correlation Between DMSs of Aging-Related Genes and Clinical Index of Asthma

**FIGURE 3:**
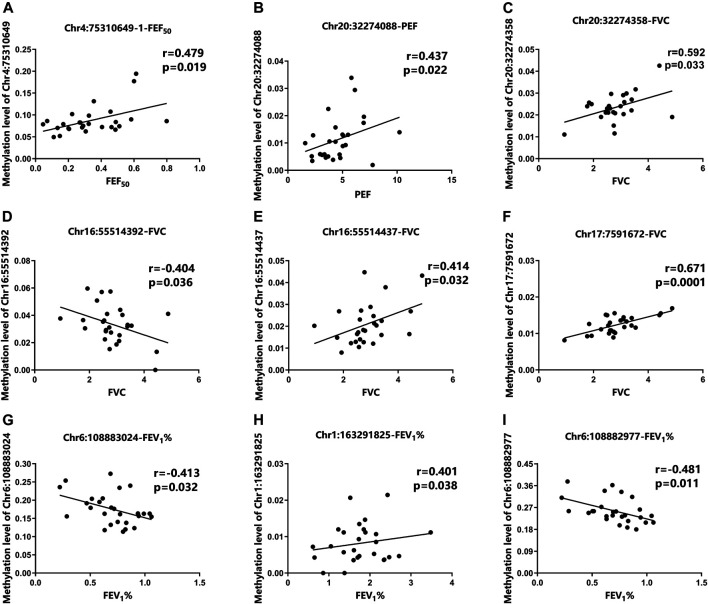
Correlation between DMSs and clinical parameters of asthma patients. **(A)** The methylation level of Chr4:75310649-1 was positively correlated with FEF_50_. **(B)** The methylation level of Chr20:32274088 was positively correlated with PEF. **(C–F)** Correlation among the methylation levels of Chr20:32274358, Chr16:55514392, Chr16:55514437, Chr17:7591672, and FVC, **(G–I)** Correlation among the methylation levels of Chr6:108883024, Chr1:163291825, and FEV_1_%.

To further assess whether the differential methylation of the nine aging-related genes is related to the occurrence and severity of asthma, we detected the correlation between the differential 68 DMSs in aging-related genes and the lung function indicators of asthma patients. The results demonstrated that nine DMSs were significantly associated with lung function. The maximum correlation coefficient for each DMS is presented in Figure 3. The remaining correlation analysis data are shown in Supplementary Figure S1. For these nine DMSs, three DMSs (Chr4:75310649-1, Chr6:108883024, and Chr17:7591672) were closely related to at least three clinical indicators. In addition, other two DMSs (Chr20:32274088 and Chr6:108882977) were related to two clinical indicators. It has also been shown that the correlation coefficients of the nine DMSs were all greater than 0.38 with a *p*-value ＜0.05. It was also particularly noteworthy that Chr17:7591672 was closely related to four lung function indicators (FVC, FEV_1_, PEF, and FEF_25_), with a correlation coefficient of 0.671 and a *p*-value equal to 0.0001. These data strongly suggested that the differential DNAm of the specific aging-related DMSs may influence the occurrence and severity of asthma. The complete data for the nine DMSs and clinical indicators are shown in Table 3.

**TABLE 3:** Correlation analysis between DNA methylation levels and clinical parameters in asthma patients.

		*p*-value							
CpG site	Gene	FEV1	FEV1%	FEV1/FVC	PEF	FVC	FEF75	FEF50	FEF25
Chr4:75310649–1	AREG	0.309	0.105	0.093	0.33	0.933	0.025^*^	0.019^*^	0.030^*^
Chr20:32274088	E2F1	0.035^*^	0.233	0.223	0.022^*^	0.051	0.05	0.05	0.068
Chr20:32274358	E2F1	0.113	0.059	0.968	0.182	0.033^*^	0.306	0.543	0.641
Chr6:108883024	FOXO3	0.044^*^	0.032^*^	0.063	0.038^*^	0.238	0.758	0.195	0.05
Chr6:108882977	FOXO3	0.063	0.011^*^	0.055	0.048^*^	0.366	0.949	0.147	0.051
Chr16:55514392	MMP2	0.064	0.243	0.424	0.104	0.036^*^	0.932	0.365	0.223
Chr16:55514437	MMP2	0.151	0.198	0.75	0.102	0.025^*^	0.343	0.489	0.246
Chr1:163291825	NUF2	0.508	0.038^*^	0.157	0.202	0.793	0.106	0.278	0.366
Chr17:7591672	TP53	0.001^*^	0.113	0.575	0.004^*^	0.0001^*^	0.758	0.171	0.019^*^

* p-value < 0.05 was considered statistically significant.

### Feasibility of Candidate DMSs as Biomarkers of Asthma

**FIGURE 4:**
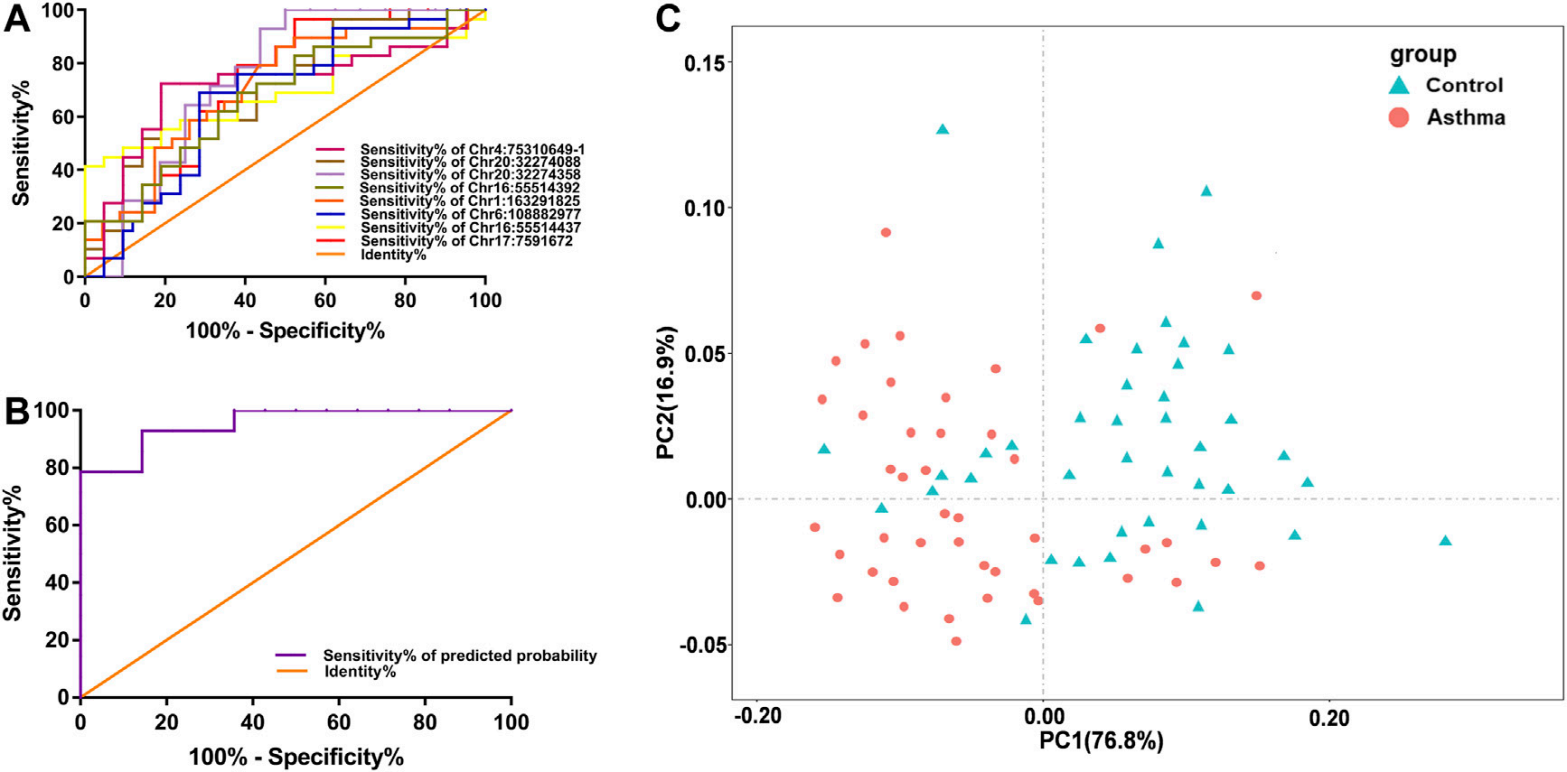
Accuracy of the methylation level of the 8 DMSs in distinguishing asthma patients from HCs. **(A)** ROC curve analysis of differential CpG sites Chr4:75310649-1, Chr20:32274088, Chr20:32274358, Chr6:108882977, Chr16:55514392, Chr16:55514437, Chr1:163291825, and Chr17:7591672, respectively. **(B)** The ROC curve of the predicted probability of the 8 DMSs. **(C)** A PCA plot consisting of the methylation levels of the 8 DMSs in HCs and asthma patients.

Since the differential nine DMSs have been confirmed to be closely associated to the clinical lung function of asthma patients, we further evaluated their potential as biomarkers for asthma patients. First, ROC analysis of the methylation levels of each candidate DMS was performed. The areas under the curve (AUC) of eight DMSs (*p*-value < 5%) were between 65.3% and 76.3%, and the AUC of six DMSs was greater than 70% (Figure 4A and Table 4). Besides, logistic regression was conducted, and the ROC of eight candidate DMSs showed that the AUC of the predicted probability of the eight candidate DMSs was as high as 95.4%, and the result was statistically significantly (*p*-value < 0.1%, Figure 4B). These results indicated that the eight candidate DMSs had the potential value to be the biomarkers for asthma. Meanwhile, to verify the above results, PCA analysis consisting of eight candidate DMSs was executed. The result revealed that the methylation levels of the total eight DMSs could effectively distinguish asthma patients from HCs (Figure 4C).

**TABLE 4:** Top nine differentially methylated sites of the differential aging-related genes associated with asthma.

CpG site	Gene	AUC	*p*-value	Optimal diagnostic threshold	Sensitivity	Specificity
Chr4:75310649-1	AREG	0.716	0.009^*^	0.086	0.724	0.81
Chr20:32274088	E2F1	0.717	0.009^*^	0.009	0.517	0.857
Chr20:32274358	E2F1	0.746	0.022^*^	0.043	1	0.533
Chr6:108883024	FOXO3	0.653	0.066	0.166	0.909	0.667
Chr6:108882977	FOXO3	0.671	0.040^*^	0.263	0.69	0.714
Chr16:55514392	MMP2	0.763	0.038^*^	0.038	0.69	0.614
Chr16:55514437	MMP2	0.688	0.024^*^	0.017	0.414	1
Chr1:163291825	NUF2	0.708	0.010^*^	0.012	0.862	0.571
Chr17:7591672	TP53	0.721	0.008^*^	0.015	0.966	0.476

Statistics were done by SPSS 22.0.

* p-value < 0.05 was considered statistically significant.

**FIGURE 5:**
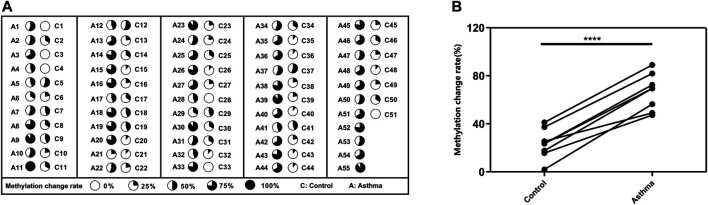
methylation change rate for asthma patients. **(A)** The methylation change rate of the 8 DMSs in asthma patients and HCs is represented by pie chart, and the dark shades indicate the percentage of the methylation change rate. **(B)** Difference in the methylation rate of single DMS in HCs and asthma patients.

**FIGURE 6:**
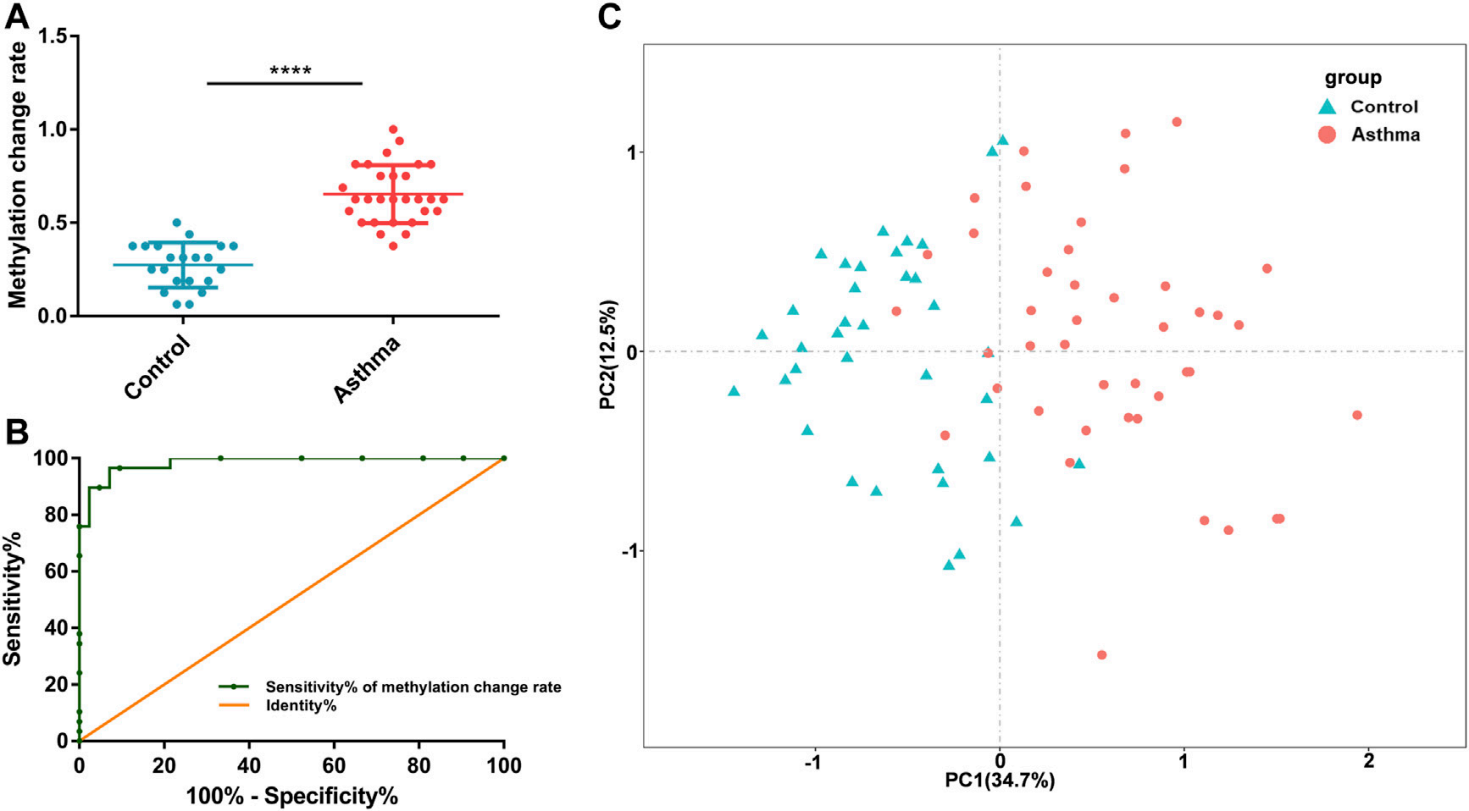
Accuracy of the 8 DMSs’ methylation change rate in distinguishing asthma patients from HCs. **(A)** Statistical analysis of the methylation change rate of the 8 DMSs in HCs and asthma patients. **(B)** ROC curve analysis of the methylation change rate in the 8 DMSs. **(C)** A PCA plot consisting of the methylation change rate of the 8 DMSs in HCs and asthma patients.

To better understand the possible value of the eight DMSs, we further calculate the methylation change rate of the eight DMSs in HCs and asthma patients, which is a description of the possibility of methylation status alteration. Then, the status of the changed methylation or unchanged methylation was determined using the optimal cutoff value. The optimal cutoffs of the eight DMSs were calculated according to the Youden index, which is presented in Table 4. The methylation change rate of HCs and asthmatic patients is included in Figure 5. Specially, the methylation change rate of the total eight DMSs in HCs showed a significant decreasing trend, whereas significantly increased methylation change rate was observed in asthma patients (Figure 5A). The methylation change rate of the total eight DMSs in asthma was 33.3–100%, and the rate in HCs was only 0–55.6%. Notably, the change rate of a single DMS in asthma patients was between 47.27% and 89.09%, while it was 1.96–41.17% in HCs (Figure 5B). Similarly, asthma patients had a higher rate of methylation change. Statistical results showed that the methylation change rate of the total eight DMSs was significantly increased in asthma patients (*p*-value < 0.1%, Figure 6A). In addition, ROC analysis was implemented according to the methylation change rate of the eight DMSs in all samples (Figure 6B), and there was a higher AUC than that in the previous method (AUC = 0.98). Moreover, the PCA analysis results also indicated that the methylation change rate of eight DMSs could better distinguish asthma patients from HCs (Figure 6C).

## Discussion

Asthma is a common chronic pulmonary disease, and the incidence of asthma has increased in the last few decades (Mazurek and Syamlal, 2018). With the increased incidence of asthma, new preventive strategies and therapies for asthma are urgently needed to further reduce the morbidity and mortality of asthma. Of particular note is the potential causal role of aging in the asthma pathogenesis (Vignola et al., 2003; Bullone and Lavoie, 2017). Several relevant studies have identified the altered expression of aging-related genes (such as TP53 and FOXO3) in respiratory diseases (Amarin et al., 2017; Hu et al., 2018). The polymorphism of transcription factor FOXO3 was confirmed to regulate the overactivation of mast cells, downregulation of anti-inflammatory factors, and production of cytokines during the pathogenesis of COPD and asthma (Barkund et al., 2015). FOXO3 deficiency has been confirmed to play an important role in regulating lung inflammation of COPD/emphysema, which has emerged as a new approach to address the development of pulmonary inflammatory diseases (Hwang et al., 2011). Similarly, TP53 has been implicated in COPD pathogens by mediating the senescence of multiple lung cells, and the overexpression of TP53 also could promote the progression of emphysema in COPD patients (Hashimoto et al., 2016; Hu et al., 2018).

Not only that, as a stable epigenetic marker, aging-related CpG sites were either hypo- or hyper-methylated in COPD and other aging-related diseases (Perez et al., 2018; Sundar and et al., 2017). Our previous research identified that DNAm was involved in regulating the expression of nine aging-related genes in peripheral venous blood of COPD patients (Du et al., 2019), as asthma and COPD have similar even overlapping clinical phenotypes in chronic inflammation and decreased lung function. In this study, we further explored the methylation change of the previous screened aging-related genes in peripheral venous blood of asthma patients. Indeed, the association between these screened nine aging-related genes and asthma has been extensively studied by previous literature works (von Bernhardi et al., 2015; Martins et al., 2016; de Sousa Neto et al., 2018; Wang S. et al., 2018; Wu and Prives, 2018; Gao et al., 2019; Huang et al., 2019; Qi et al., 2019). AREG, E2F1, FOXO3, HDAC1, MMP2, TGFB1, and TP53 have been confirmed as crucial signaling molecules in asthma (Enomoto et al., 2009; Nakagome and Nagata, 2011; Butler et al., 2012; Xu, 2014; Toujani et al., 2016; Amarin et al., 2017; Hur and Broide, 2019; Wang et al., 2019). Although ATG3 is a key central regulator in autophagy induction during aging (Frudd et al., 2018), and NUF2 is closely associated with lung cell senescence (Xing et al., 2016), their specific role in asthma has rarely been studied. The differential expression of ATG3, FOXO3, NUF2, and TP53 in asthma patients was also aligned with that in former studies (Xuan and Hou, 2014; Xing et al., 2016; Amarin et al., 2017; Tsai et al., 2019). In addition, excessive secretion of AREG in the airway after acute asthma attack promotes airway remodeling (Enomoto et al., 2009). However, AREG is downregulated in peripheral blood of elderly asthma patients, which may be attributed to the different disease stages. It is particularly worth noting that the decreased expression of E2F1 in asthma patients is consistent with what we have previously observed in COPD patients (Du et al., 2019), which is different from that in lung cancer patients (Tsai et al., 2019). One possible reason is the specificity of the sample tissue and pathogenic genes in different diseases. MMP2, as a member of the matrix metalloproteinase family, shows an increasing trend in the acute and chronic phases of lung disease. Our results observed the increased expression of MMP2 in asthma patients, which is consistent with that in previous literature (Greenlee et al., 2007).

Additionally, we identified the methylation status of the nine aging-related genes in asthma patients. Most DMSs of asthma patients were hypermethylated, which was consistent with the differential expression of mRNA, indicating that DNAm-regulating gene expression is related to aging. Moreover, except for ATG3, HDAC1, and TGFB1, correlation analysis showed that the expression of the aging-related genes in peripheral blood of asthma patients was associated with pulmonary function parameters (FEV_1_%, FEV_1_, FVC, PEF, FEF_75_, FEF_50_, and FEF_25_). It is known that TGFB1 was a key cytokine that directs airway remodeling (Dragicevic et al., 2016), and HDAC1 played a critical role in the pathogenesis of asthma (Wang C. et al., 2018). This partial difference may be due to the presence of single-nucleotide polymorphism in asthma (Shan et al., 2018). Chr16:55514392 located in the promoter region has a regulatory effect on gene expression, which is inversely associated with the lung function index (FVC) (Haberle and Stark, 2018). Interestingly, Chr16:55514437 is also located at the transcription initiation site, but the specific molecular mechanism which regulates gene expression still needs further study (Haberle and Stark, 2018). Furthermore, there were eight asthma-related CpG sites on the CpG islands of the differential aging-related genes. The ROC curve and PCA analysis of the methylation level showed that all the eight DMSs could be used as potential biomarkers to distinguish asthma from HCs. Most notably, the methylation rate of either single DMS or total eight DMSs in asthma patients was significantly higher than that of HCs. As population size and ethnicity may influence the methylation level, we assumed that a methylation marker hold promise for better biomarker of asthma. Previous studies have shown that the decreased methylation level of the promoter region regulates the proliferation of asthmatic airway smooth muscle cells, which is related to the severity of asthma and can be used a potential biomarker for predicting asthma exacerbation (Perry et al., 2018). In addition, it has also been pointed out that the methylation levels of FOXO3 and TP53 can be used as biomarker targets for late-onset asthma (Yuan et al., 2020). Our analysis of the eight DMSs’ methylation mutation rate also produced a better ROC specificity and sensitivity, suggesting that the combinatorial DMSs had a great potential to predict asthma. BALF (IL-25, IL-33, *etc*.), induced sputum (eosinophils, Th2 cells, *etc*.), and airway remodeling could all be used as a useful indicator for asthma diagnosis (Lefaudeux et al., 2017; Li et al., 2018). However, the detection of DNAm in peripheral blood has greater advantage of widespread access to samples and simple operation. Compared with other clinical biomarkers, such as blood eosinophils, exhaled nitric oxide (FeNO), and sputum eosinophils (Fitzpatrick and Moore, 2017), detection of DNAm in peripheral blood has some clear advantages. Although sputum eosinophils have been the “gold standard” Type-2 inflammatory biomarker (Coumou and Bel, 2016), performing sputum analysis in clinic is still risky to some extent. Besides, exhaled nitric oxide (FeNO) has a relatively large individual difference. However, detection of DNAm has greater clinical feasibility which is noninvasive and cost-effective. Not only that, DNAm is also an important cause of asthma exacerbation; the specific role of allergens and environmental exposure on the epigenetic modification during the exacerbation of asthma also deserved more attention (Bae et al., 2020).

Although our study provides potential value for diagnosis and treatment of asthma assessment, there are also some limitations. First, asthma can be divided into different phenotypes which may have differential epigenetic modification. Besides, our previous work is not comprehensive enough to screen all the aging-related genes. Moreover, the sample size is relatively small.

## Conclusion

In a word, this study demonstrated that DNAm may regulate the differential mRNA expression of aging-related genes in the peripheral blood of asthma patients. Besides, the specific DMSs in aging-related genes have been strongly associated with the pulmonary function index of asthma patients. These results shed new light on DNAm that may be involved in regulating aging-related genes in asthma, which may also provide potential candidate biomarkers for the early diagnosis of asthma.

## Data Availability

The raw data supporting the conclusions of this article will be made available by the authors, without undue reservation.

## Supplementary Material

SUPPLEMENTARY FIGURE S1 | Correlation analysis between differential methylation sites and clinical parameters of asthma patients. **(A, B)** The methylation levels of Chr4:75310649-1 and Chr17:7591672 were positively correlated with those of PEF_25_. **(C)** The methylation level of Chr4:75310649-1 was positively correlated with that of FEF_75_. **(D–F)** Correlation between the methylation levels of Chr20:32274088, Chr6:108883024, and Chr17:7591672, and FEV_1_. **(G–I)** Correlation between the methylation levels of Chr6:108883024, Chr6:108882977, and Chr17:7591672, and PEF.

SUPPLEMENTARY TABLE S1 | The data of 68 CpG sites related to DNAm.

SUPPLEMENTARY TABLE S2 | The correlation between mRNA expression and DNAm of 68 CpG sites of the 9 aging-related genes.

## References

[B1] Aghasafari P. George U. Pidaparti R. (2019). A Review of Inflammatory Mechanism in Airway Diseases. Inflamm. Res. 68 (1), 59–74. 10.1007/s00011-018-1191-2 30306206

[B2] Amarin J. Z. Naffa R. G. Suradi H. H. Alsaket Y. M. Obeidat N. M. Mahafza T. M.(2017). An Intronic Single-Nucleotide Polymorphism (Rs13217795) in FOXO3 Is Associated with Asthma and Allergic Rhinitis: a Case-Case-Control Study. BMC Med. Genet. 18 (1), 132. 10.1186/s12881-017-0494-4 29141605 PMC5688628

[B3] Bae D.-J. Jun J. A. Chang H. S. Park J. S. Park C.-S. (2020). Epigenetic Changes in Asthma: Role of DNA CpG Methylation. Tuberc. Respir. Dis. 83 (1), 1–13. 10.4046/trd.2018.0088 PMC6953489 31905427

[B4] Barkund S. Kantamneni H. Donzanti M. Martin D. Zhao X. He S. (2015). FOXO3a Gene Polymorphism Associated with Asthma in Indian Population. Mol. Biol. Int. 2015, 638515. 10.1155/2015/638515 26783460 PMC4689967

[B5] Budde J. Skloot G. S. (2018). Is Aging a "comorbidity" of Asthma?. Pulm. Pharmacol. Ther. 52, 52–56. 10.1016/j.pupt.2018.06.005 29981459

[B6] Bullone M. Lavoie J. P. (2017). The Contribution of Oxidative Stress and Inflamm-Aging in Human and Equine Asthma. Int. J. Mol. Sci. 18 (12). 10.3390/ijms18122612 PMC5751215 29206130

[B7] Butler C. A. McQuaid S. Taggart C. C. Weldon S. Carter R. Skibinski G. (2012). Glucocorticoid Receptor β and Histone Deacetylase 1 and 2 Expression in the Airways of Severe Asthma. Thorax 67 (5), 392–398. 10.1136/thoraxjnl-2011-200760 22156779

[B8] Conte E. Fagone E. Fruciano M. Gili E. Iemmolo M. Vancheri C. (2015). Anti-inflammatory and Antifibrotic Effects of Resveratrol in the Lung. Histol. Histopathol 30 (5), 523–529. 10.14670/HH-30.523 25515609

[B9] Coumou H. Bel E. H. (2016). Improving the Diagnosis of Eosinophilic Asthma. Expert Rev. Respir. Med. 10 (10), 1093–1103. 10.1080/17476348.2017.1236688 27624868

[B10] de Sousa Neto I. V. Durigan J. L. Q. Guzzoni V. Tibana R. A. Prestes J. de Araujo H. S. S. (2018). Effects of Resistance Training on Matrix Metalloproteinase Activity in Skeletal Muscles and Blood Circulation During Aging. Front. Physiol. 9, 190. 10.3389/fphys.2018.00190 29593554 PMC5857587

[B11] DeVries A. Vercelli D. (2016). Epigenetic Mechanisms in Asthma. Ann. Am. Thorac. Soc. 13 Suppl 1 (Suppl. 1Suppl 1), S48–S50. 10.1513/AnnalsATS.201507-420MG 27027952 PMC5015730

[B12] Dragicevic S. Petrovic-Stanojevic N. Nikolic A. (2016). TGFB1 Gene Promoter Polymorphisms in Serbian Asthmatics. Adv. Clin. Exp. Med. 25 (2), 273–278. 10.17219/acem/32211 27627560

[B13] Du X. Yuan L. Wu M. Men M. He R. Wang L. (2019). Variable DNA Methylation of Aging-Related Genes Is Associated with Male COPD. Respir. Res. 20 (1), 243. 10.1186/s12931-019-1215-7 31684967 PMC6829949

[B14] Dunn R. M. Busse P. J. Wechsler M. E. (2018). Asthma in the Elderly and Late-Onset Adult Asthma. Allergy 73 (2), 284–294. 10.1111/all.13258 28722758

[B15] Enomoto Y. Orihara K. Takamasu T. Matsuda A. Gon Y. Saito H. (2009). Tissue Remodeling Induced by Hypersecreted Epidermal Growth Factor and Amphiregulin in the Airway after an Acute Asthma Attack. J. Allergy Clin. Immunol. 124 (5), 913–920. 10.1016/j.jaci.2009.08.044 19895983

[B16] Field A. E. Robertson N. A. Wang T. Havas A. Ideker T. Adams P. D. (2018). DNA Methylation Clocks in Aging: Categories, Causes, and Consequences. Mol. Cel 71 (6), 882–895. 10.1016/j.molcel.2018.08.008 PMC6520108 30241605

[B17] Fitzpatrick A. M. Moore W. C. (2017). Severe Asthma Phenotypes - How Should They Guide Evaluation and Treatment?. J. Allergy Clin. Immunol. Pract. 5 (4), 901–908. 10.1016/j.jaip.2017.05.015 28689840 PMC5541906

[B18] Fluss R. Faraggi D. Reiser B. (2005). Estimation of the Youden Index and its Associated Cutoff Point. Biom. J. 47 (4), 458–472. 10.1002/bimj.200410135 16161804

[B19] Frudd K. Burgoyne T. Burgoyne J. R. (2018). Oxidation of Atg3 and Atg7 Mediates Inhibition of Autophagy. Nat. Commun. 9 (1), 95. 10.1038/s41467-017-02352-z 29311554 PMC5758830

[B20] Gao S. Song Q. Liu J. Zhang X. Ji X. Wang P. (2019). E2F1 Mediates the Downregulation of POLD1 in Replicative Senescence. Cell. Mol. Life Sci. 76 (14), 2833–2850. 10.1007/s00018-019-03070-z 30895337 PMC6588650

[B21] Gina Report (2021). Global Strategy for Asthma Management and Prevention. Available from:https://ginasthma.org/gina-reports/ (Accessed April 29, 2021).

[B22] Greenlee K. J. Werb Z. Kheradmand F. (2007). Matrix Metalloproteinases in Lung: Multiple, Multifarious, and Multifaceted. Physiol. Rev. 87 (1), 69–98. 10.1152/physrev.00022.2006 17237343 PMC2656382

[B23] Haberle V. Stark A. (2018). Eukaryotic Core Promoters and the Functional Basis of Transcription Initiation. Nat. Rev. Mol. Cel Biol 19 (10), 621–637. 10.1038/s41580-018-0028-8 PMC6205604 29946135

[B24] Hashimoto Y. Sugiura H. Togo S. Koarai A. Abe K. Yamada M. (2016). 27-Hydroxycholesterol Accelerates Cellular Senescence in Human Lung Resident Cells. Am. J. Physiology-Lung Cell Mol. Physiol. 310 (11), L1028–L1041. 10.1152/ajplung.00351.2015 27036870

[B25] Hu W.-p. Zeng Y.-y. Zuo Y.-h. Zhang J. (2018). Identification of Novel Candidate Genes Involved in the Progression of Emphysema by Bioinformatic Methods. Copd 13, 3733–3747. 10.2147/copd.s183100 PMC6241693 30532529

[B26] Huang W. Zeng C. Hu S. Wang L. Liu J. (2019). ATG3, a Target of miR-431-5p, Promotes Proliferation and Invasion of Colon Cancer *via* Promoting Autophagy. Cmar 11, 10275–10285. 10.2147/cmar.s226828 PMC6911302 31849517

[B27] Hur G. Y. Broide D. H. (2019). Genes and Pathways Regulating Decline in Lung Function and Airway Remodeling in Asthma. Allergy Asthma Immunol. Res. 11 (5), 604–621. 10.4168/aair.2019.11.5.604 31332973 PMC6658410

[B28] Hwang J.-w. Rajendrasozhan S. Yao H. Chung S. Sundar I. K. Huyck H. L. (2011). FOXO3 Deficiency Leads to Increased Susceptibility to Cigarette Smoke-Induced Inflammation, Airspace Enlargement, and Chronic Obstructive Pulmonary Disease. J.I. 187 (2), 987–998. 10.4049/jimmunol.1001861 PMC3131437 21690325

[B29] Johnson A. A. Akman K. Calimport S. R. G. Wuttke D. Stolzing A. de Magalhães J. P. (2012). The Role of DNA Methylation in Aging, Rejuvenation, and Age-Related Disease. Rejuvenation Res. 15 (5), 483–494. 10.1089/rej.2012.1324 23098078 PMC3482848

[B30] Kennedy B. K. Berger S. L. Brunet A. Campisi J. Cuervo A. M. Epel E. S. (2014). Geroscience: Linking Aging to Chronic Disease. Cell 159 (4), 709–713. 10.1016/j.cell.2014.10.039 25417146 PMC4852871

[B31] Koshy L. Anju A. L. Harikrishnan S. Kutty V. R. Jissa V. T. Kurikesu I. (2017). Evaluating Genomic DNA Extraction Methods from Human Whole Blood Using Endpoint and Real-Time PCR Assays. Mol. Biol. Rep. 44 (1), 97–108. 10.1007/s11033-016-4085-9 27686559

[B32] Lefaudeux D. De Meulder B. Loza M. J. Peffer N. Rowe A. Baribaud F. (2017). U-BIOPRED Clinical Adult Asthma Clusters Linked to a Subset of Sputum Omics. J. Allergy Clin. Immunol. 139 (6), 1797–1807. 10.1016/j.jaci.2016.08.048 27773852

[B33] Li J.-J. Li S. Zhu C.-G. Wu N.-Q. Zhang Y. Guo Y.-L. (2017). Familial Hypercholesterolemia Phenotype in Chinese Patients Undergoing Coronary Angiography. Arterioscler Thromb. Vasc. Biol. 37 (3), 570–579. 10.1161/atvbaha.116.308456 27932355

[B34] Li Y. Wang W. Lv Z. Li Y. Chen Y. Huang K. (2018). Elevated Expression of IL-33 and TSLP in the Airways of Human Asthmatics *In Vivo*: A Potential Biomarker of Severe Refractory Disease. J.I. 200 (7), 2253–2262. 10.4049/jimmunol.1701455 29453280

[B35] Luhadia S. K. (2014). Steroid Resistant Asthma. J. Assoc. Physicians India 62 (3 Suppl. l), 38–40. 25327059

[B36] Maltby S. Tay H. L. Yang M. Foster P. S. (2017). Mouse Models of Severe Asthma: Understanding the Mechanisms of Steroid Resistance, Tissue Remodelling and Disease Exacerbation. Respirology 22 (5), 874–885. 10.1111/resp.13052 28401621

[B37] Martins R. Lithgow G. J. Link W. (2016). Long Live FOXO : Unraveling the Role of FOXO Proteins in Aging and Longevity. Aging Cell 15 (2), 196–207. 10.1111/acel.12427 26643314 PMC4783344

[B38] Mazurek J. M. Syamlal G. (2018). Prevalence of Asthma, Asthma Attacks, and Emergency Department Visits for Asthma Among Working Adults - National Health Interview Survey, 2011-2016. MMWR Morb. Mortal. Wkly. Rep. 67 (13), 377–386. 10.15585/mmwr.mm6713a1 29621204 PMC5889242

[B39] Miller R. L. Lawrence J. (2018). Understanding Root Causes of Asthma. Perinatal Environmental Exposures and Epigenetic Regulation. Ann. ATS 15 (Suppl. 2), S103–s108. 10.1513/annalsats.201706-514mg PMC5946504 29676631

[B40] Miravitlles M. Guerrero T. Mayordomo C. Sánchez-Agudo L. Nicolau F. Segú J. L. (2000). Factors Associated with Increased Risk of Exacerbation and Hospital Admission in a Cohort of Ambulatory COPD Patients: a Multiple Logistic Regression Analysis. Respiration 67 (5), 495–501. 10.1159/000067462 11070451

[B41] Morales-Nebreda L. McLafferty F. S. Singer B. D. (2019). DNA Methylation as a Transcriptional Regulator of the Immune System. Translational Res. 204, 1–18. 10.1016/j.trsl.2018.08.001 PMC6331288 30170004

[B42] Morrow J. D. Cho M. H. Hersh C. P. Pinto-Plata V. Celli B. Marchetti N. (2016). DNA Methylation Profiling in Human Lung Tissue Identifies Genes Associated with COPD. Epigenetics 11 (10), 730–739. 10.1080/15592294.2016.1226451 27564456 PMC5094634

[B43] Morrow J. D. Glass K. Cho M. H. Hersh C. P. Pinto-Plata V. Celli B. (2018). Human Lung DNA Methylation Quantitative Trait Loci Colocalize with Chronic Obstructive Pulmonary Disease Genome-wide Association Loci. Am. J. Respir. Crit. Care Med. 197 (10), 1275–1284. 10.1164/rccm.201707-1434oc 29313708 PMC5955059

[B44] Nakagome K. Nagata M. (2011). Pathogenesis of Airway Inflammation in Bronchial Asthma. Auris Nasus Larynx 38 (5), 555–563. 10.1016/j.anl.2011.01.011 21334836

[B45] Nicodemus-Johnson J. Myers R. A. Sakabe N. J. Sobreira D. R. Hogarth D. K. Naureckas E. T. (2016). DNA Methylation in Lung Cells Is Associated with Asthma Endotypes and Genetic Risk. JCI Insight 1 (20), e90151. 10.1172/jci.insight.90151 27942592 PMC5139904

[B46] Peng C. Cardenas A. Rifas-Shiman S. L. Hivert M.-F. Gold D. R. Platts-Mills T. A. (2019). Epigenetic Age Acceleration Is Associated with Allergy and Asthma in Children in Project Viva. J. Allergy Clin. Immunol. 143 (6), 2263–2270. 10.1016/j.jaci.2019.01.034 30738172 PMC6556426

[B47] Perez R. F. Tejedor J. R. Bayón G. F. Fernández A. F. Fraga M. F. (2018). Distinct Chromatin Signatures of DNA Hypomethylation in Aging and Cancer. Aging Cell 17 (3), e12744. 10.1111/acel.12744 29504244 PMC5946083

[B48] Perry M. M. Lavender P. Kuo C. S. Galea F. Michaeloudes C. Flanagan J. M. (2018). DNA Methylation Modules in Airway Smooth Muscle Are Associated with Asthma Severity. Eur. Respir. J. 51 (4). 10.1183/13993003.01068-2017 PMC5902304 29449426

[B49] Qi C. Xu C.-J. Koppelman G. H. (2019). The Role of Epigenetics in the Development of Childhood Asthma. Expert Rev. Clin. Immunol. 15 (12), 1287–1302. 10.1080/1744666x.2020.1686977 31674254

[B50] Saito H. Miyatani K. Kono Y. Murakami Y. Kuroda H. Matsunaga T. (2017). Decreased Serum Concentration of Total IgG Is Related to Tumor Progression in Gastric Cancer Patients. Yonago Acta Med. 60 (2), 119–125. 10.33160/yam.2017.06.008 28701895 PMC5502224

[B51] Shan L. Hou P. Kang X. Shang Y. (2018). Effects of Single-Nucleotide Polymorphisms in the TLR7 and TLR9 Genes of Asthmatic Children. Ann. Clin. Lab. Sci. 48 (5), 601–607. 30373864

[B52] Sundar I. K. Yin Q. Baier B. S. Yan L. Mazur W. Li D. (2017). DNA Methylation Profiling in Peripheral Lung Tissues of Smokers and Patients with COPD. Clin. Epigenetics 9, 38. 10.1186/s13148-017-0335-5 28416970 PMC5391602

[B53] Toujani S. Mehiri N. Hamzaoui K. Hadded H. Ben Salah N. Mjid M. (2016). Role of Metalloproteinases MMP-2 in Asthma. Tunis Med. 94 (6), 167–171. 28051223

[B54] Tsai M. J. Tsai Y. C. Chang W. A. Lin Y. S. Tsai H. Sheu C. C. (2019). Deducting MicroRNA-Mediated Changes Common in Bronchial Epithelial Cells of Asthma and Chronic Obstructive Pulmonary Disease-A Next-Generation Sequencing-Guided Bioinformatic Approach. Int. J. Mol. Sci. 20 (3), 553. 10.3390/ijms20030553 PMC6386886 30696075

[B55] Vignola A. M. Scichilone N. Bousquet J. Bonsignore G. Bellia V. (2003). Aging and Asthma: Pathophysiological Mechanisms. Allergy 58 (3), 165–175. 10.1034/j.1398-9995.2003.02163.x 12653790

[B56] von Bernhardi R. Eugenin-von Bernhardi L. Eugenin J. (2015). Microglial Cell Dysregulation in Brain Aging and Neurodegeneration. Front. Aging Neurosci. 7, 124. 10.3389/fnagi.2015.00124 26257642 PMC4507468

[B59] Wang C. Li H. Cao L. Wang G. (2018). Identification of Differentially Expressed Genes Associated with Asthma in Children Based on the Bioanalysis of the Regulatory Network. Mol. Med. Rep. 18 (2), 2153–2163. 10.3892/mmr.2018.9205 29956778 PMC6072229

[B58] Wang S. Ge W. Harns C. Meng X. Zhang Y. Ren J. (2018). Ablation of Toll-like Receptor 4 Attenuates Aging-Induced Myocardial Remodeling and Contractile Dysfunction through NCoRI-HDAC1-Mediated Regulation of Autophagy. J. Mol. Cell Cardiol. 119, 40–50. 10.1016/j.yjmcc.2018.04.009 29660306

[B60] Wang J. Zhu M. Wang L. Chen C. Song Y. (2019). Amphiregulin Potentiates Airway Inflammation and Mucus Hypersecretion Induced by Urban Particulate Matter *via* the EGFR-Pi3kα-AKT/ERK Pathway. Cell Signal. 53, 122–131. 10.1016/j.cellsig.2018.10.002 30291869

[B57] Wang Z. N. Su R. N. Yang B. Y. Yang K. X. Yang L. F. Yan Y. (2020). Potential Role of Cellular Senescence in Asthma. Front Cel Dev Biol 8, 59. 10.3389/fcell.2020.00059 PMC7026390 32117985

[B61] Wu D. Prives C. (2018). Relevance of the P53-MDM2 axis to Aging. Cell Death Differ 25 (1), 169–179. 10.1038/cdd.2017.187 29192902 PMC5729541

[B62] Xing Y. Zhang J. Lu L. Li D. Wang Y. Huang S. (2016). Identification of Hub Genes of Pneumocyte Senescence Induced by Thoracic Irradiation Using Weighted Gene Co-expression Network Analysis. Mol. Med. Rep. 13 (1), 107–116. 10.3892/mmr.2015.4566 26572216 PMC4686054

[B63] Xu W. (2014). Expression Data Analysis to Identify Biomarkers Associated with Asthma in Children. Int. J. Genomics 2014, 165175. 10.1155/2014/165175 24790987 PMC3985200

[B64] Xuan L. L. Yao X. Xue B. Hou Q. (2014). [Recent Advances in the Study of AMPK and Inflammatory Pulmonary Disease], 49 (8), 1089–1096. 25322548

[B65] Yang I. V. Pedersen B. S. Rabinovich E. Hennessy C. E. Davidson E. J. Murphy E. (2014). Relationship of DNA Methylation and Gene Expression in Idiopathic Pulmonary Fibrosis. Am. J. Respir. Crit. Care Med. 190 (11), 1263–1272. 10.1164/rccm.201408-1452oc 25333685 PMC4315819

[B67] Yuan L. Du X. Tang S. Wu S. Wang L. Xiang Y. (2019). ITGB 4 Deficiency Induces Senescence of Airway Epithelial Cells through P53 Activation. Febs j 286 (6), 1191–1203. 10.1111/febs.14749 30636108

[B66] Yuan L. Wang L. Du X. Qin L. Yang M. Zhou K. (2020). The DNA Methylation of FOXO3 and TP53 as a Blood Biomarker of Late-Onset Asthma. J. Transl Med. 18 (1), 467. 10.1186/s12967-020-02643-y 33298101 PMC7726856

[B68] Yuan L. Zhang X. Yang M. Du X. Wang L. Wu S. (2020). Airway Epithelial Integrin β4 Suppresses Allergic Inflammation by Decreasing CCL17 Production. Clin. Sci. (Lond) 134 (13), 1735–1749. 10.1042/cs20191188 32608482

[B69] Zhou-Suckow Z. Duerr J. Hagner M. Agrawal R. Mall M. A. (2017). Airway Mucus, Inflammation and Remodeling: Emerging Links in the Pathogenesis of Chronic Lung Diseases. Cell Tissue Res 367 (3), 537–550. 10.1007/s00441-016-2562-z 28108847

[B70] Zhu H. Wang G. Qian J. (2016). Transcription Factors as Readers and Effectors of DNA Methylation. Nat. Rev. Genet. 17 (9), 551–565. 10.1038/nrg.2016.83 27479905 PMC5559737

