# Supplementary material for: Aberrant Methylation of Aging-Related Genes in Asthma
Source: Front Mol Biosci. 2021 May 25;8:655285. doi: 10.3389/fmolb.2021.655285 (PMC8203316; doi:10.3389/fmolb.2021.655285)
Supplement: Supplementary file 1 [file Table1.DOCX]

**Table 1**. Primer sequence of aging-related genes for qPCR.

| Gene | Primer |  |
| --- | --- | --- |
| AREG | forward | TGTCGCTCTTGATACTCGGC |
|  | reverse | AGGCATTTCACTCACAGGGG |
| ATG3 | forward | GTGTTCAGTTCACCCATGCAG |
|  | reverse | TTAACAGCCATTTTGCCACTAATCT |
| E2F1 | forward | CATCCCAGGAGGTCACTTCTG |
|  | reverse | GACAACAGCGGTTCTTGCTC |
| FOXO3 | forward | CGGACAAACGGCTCACTCT |
|  | reverse | GGACCCGCATGAATCGACTAT |
| HDAC1 | forward | TTTTTGGGTYGGAYGTTGAG |
|  | reverse | CCCTCRCAACCTCCTCTCC |
| MMP2 | forward | TGGCACCCATTTACACCTAC |
|  | reverse | CCTCGTATACCGCATCAATC |
| NUF2 | forward | TGTTAAGCAATACAAACGCACAG |
|  | reverse | TGCCTTTTCAATACCGTCGTG |
| TGFB1 | forward | CGACTCGCCAGAGTGGTTAT |
|  | reverse | GCTAAGGCGAAAGCCCTCAA |
| TP53 | forward | AAGTCTGTGACTTGCACGTACTCC |
|  | reverse | GTCATGTGCTGTGACTGCTTGTAG |
| β-actin | forward | TTCCAGCCTTCCTTCCTGGG |
|  | reverse | TTGCGCTCAGGAGGAGCAAT |
