# Supplementary material for: Aberrant Methylation of Aging-Related Genes in Asthma
Source: Front Mol Biosci. 2021 May 25;8:655285. doi: 10.3389/fmolb.2021.655285 (PMC8203316; doi:10.3389/fmolb.2021.655285)
Supplement: Supplementary file 3 [file Table2.DOCX]

**Table 2**. Demographic characteristics of asthma patients and HCs.

|  | Control | Asthma |
| --- | --- | --- |
| Number of subjects | 51 | 55 |
| Age | 53.83±6.84 | 46.72±10.41 |
| Gender (f/m) | 41/10 | 46/9 |
| FEV_1_ | 2.82±0.20 | 1.76±0.62* |
| FEV_1_ % predicted | 0.92±0.25 | 0.70±0.24* |
| FVC | 4.02±0.65 | 2.84±0.85* |
| FEV_1_/FVC | 0.83±0.03 | 0.65±0.16* |
| PEF | 8.34±0.92 | 4.65±1.84* |
| FEF_75_ | 0.83±0.35 | 0.47±0.22* |
| FEF_50_ | 0.82±0.34 | 0.37±0.16* |
| FEF_25_ | 0.72±0.22 | 0.23±0.18* |

Data are presented as Mean ± SD. **p*-value < 0.05, asthma patients VS controls (Unpaired t test).
