# Supplementary material for: Aberrant Methylation of Aging-Related Genes in Asthma
Source: Front Mol Biosci. 2021 May 25;8:655285. doi: 10.3389/fmolb.2021.655285 (PMC8203316; doi:10.3389/fmolb.2021.655285)
Supplement: Supplementary file 4 [file Table5.DOCX]

**Table S1.** The data of 68 CpG sites related to DNAm.

| CpG site | Gene | Mean Difference Methylation | *p*-value |
| --- | --- | --- | --- |
| **Chr4:75310759-1** | AREG | -0.0028 | 0.0150* |
| **Chr4:75310759-2** | AREG | -0.0022 | 0.0350* |
| **Chr4:75310759-3** | AREG | 0.0065 | 0.0090* |
| **Chr4:75310740-1** | AREG | -0.0122 | 0.0090* |
| **Chr4:75310740-2** | AREG | -0.0027 | 0.0380* |
| **Chr4:75310649-1** | AREG | -0.0050 | 0.0250* |
| **Chr4:75310649-2** | AREG | 0.0034 | 0.0380* |
| **Chr4:75480850** | AREG | -0.0021 | 0.0400* |
| **Chr4:75311007** | AREG | 0.0031 | 0.0280* |
| **Chr4:75310861** | AREG | -0.0015 | 0.0090* |
| **Chr3:112280603** | ATG3 | -0.0022 | 0.0030* |
| **Chr20:32274084** | E2F1 | -0.0055 | 0.0430* |
| **Chr20:32274088** | E2F1 | 0.0054 | 0.0090* |
| **Chr20:32274171** | E2F1 | 0.0052 | 0.0240* |
| **Chr20:32274205** | E2F1 | 0.0037 | 0.0450* |
| **Chr20:32274325** | E2F1 | -0.0076 | 0.0340* |
| **Chr20:32274351** | E2F1 | 0.0198 | 0.0140* |
| **Chr20:32274358-1** | E2F1 | -0.0202 | 0.0230* |
| **Chr20:32274358-2** | E2F1 | -0.0094 | 0.0160* |
| **Chr20:32274325** | E2F1 | -0.0169 | 0.0030* |
| **Chr20:32274387** | E2F1 | 0.0364 | 0.0100* |
| **Chr6:108879441** | FOXO3 | -0.0036 | 0.0010* |
| **Chr6:108879379** | FOXO3 | -0.0007 | 0.0221* |
| **Chr6:108879141** | FOXO3 | -0.0012 | 0.0290* |
| **Chr6:108879095** | FOXO3 | 0.0008 | 0.0210* |
| **Chr6:108879086** | FOXO3 | 0.0018 | 0.0400* |
| **Chr6:108879922** | FOXO3 | -0.0017 | 0.0430* |
| **Chr6:108880271** | FOXO3 | -0.0009 | 0.0050* |
| **Chr6:108883024** | FOXO3 | -0.0987 | 0.0430* |
| **Chr6:108882982** | FOXO3 | -0.1174 | 0.0250* |
| **Chr6:108882977** | FOXO3 | -0.1142 | 0.0230* |
| **Chr6:108882964** | FOXO3 | -0.110 | 0.0380* |
| **Chr6:108882941** | FOXO3 | -0.1123 | 0.0410* |
| **Chr6:108882900** | FOXO3 | -0.1307 | 0.0250* |
| **Chr6:108882898** | FOXO3 | -0.1286 | 0.0380* |
| **Chr6:108882825** | FOXO3 | -0.1070 | 0.0340* |
| **Chr6:108882816** | FOXO3 | -0.0976 | 0.0350* |
| **Chr6:108881593** | FOXO3 | 0.0000 | 0.0390* |
| **Chr6:108881542** | FOXO3 | 0.0000 | 0.0300* |
| **Chr1:32757756** | HDAC1 | 0.0042 | 0.0380* |
| **Chr1:32757781** | HDAC1 | -0.0036 | 0.0470* |
| **Chr16:55512764** | MMP2 | -0.0182 | 0.0080* |
| **Chr16:55513222** | MMP2 | -0.0032 | 0.0230* |
| **Chr16:55513192** | MMP2 | -0.0085 | 0.0320* |
| **Chr16:55514313** | MMP2 | -0.0088 | 0.0070* |
| **Chr16:55514392** | MMP2 | -0.0095 | 0.0200* |
| **Chr16:55514399** | MMP2 | -0.0114 | 0.0430* |
| **Chr16:55514437** | MMP2 | -0.0043 | 0.0240* |
| **Chr16:55514439** | MMP2 | -0.0038 | 0.0260* |
| **Chr16:55514462** | MMP2 | -0.0136 | 0.0340* |
| **Chr16:55514466** | MMP2 | -0.0138 | 0.0230* |
| **Chr16:55514468** | MMP2 | -0.0159 | 0.0020* |
| **Chr16:55514470** | MMP2 | -0.0132 | 0.0020* |
| **Chr1:163291676** | NUF2 | -0.0035 | 0.0190* |
| **Chr1:163291713** | NUF2 | -0.0024 | 0.0300* |
| **Chr1:163291825** | NUF2 | -0.0041 | 0.0150* |
| **Chr1:163291828** | NUF2 | -0.0045 | 0.0270* |
| **Chr1:163291897** | NUF2 | -0.0070 | 0.0260* |
| **Chr19:41859711** | TGFB1 | 0.0178 | 0.0340* |
| **Chr19:41859607-1** | TGFB1 | -0.0079 | 0.0300* |
| **Chr19:41859607-2** | TGFB1 | -0.0048 | 0.0140* |
| **Chr19:41858716** | TGFB1 | -0.0043 | 0.0310* |
| **Chr19:41858683** | TGFB1 | -0.0038 | 0.0090* |
| **Chr19:41858034** | TGFB1 | -0.0026 | 0.0240* |
| **Chr17:7591672** | TP53 | -0.0028 | 0.0090* |
| **Chr17:7591565** | TP53 | 0.0000 | 0.0430* |
| **Chr17:7591520** | TP53 | 0.0026 | 0.0120* |
| **Chr17:7590743** | TP53 | -0.0011 | 0.0120* |

Differential methylation analysis was conducted between asthma patients and controls in blood samples from a total of 106 subjects. The method of Benjamin Hochberg was used to control the false discovery rate (FDR), * *p*-value<0.05. Several sequencing data of the same CpG site are distinguished by numbers. e.g. Chr4:75310649-1, Chr4:75310649-2，Chr4:75310759-3.
