# Supplementary material for: Aberrant Methylation of Aging-Related Genes in Asthma
Source: Front Mol Biosci. 2021 May 25;8:655285. doi: 10.3389/fmolb.2021.655285 (PMC8203316; doi:10.3389/fmolb.2021.655285)
Supplement: Supplementary file 5 [file Table3.DOCX]

**Table 3.** Correlation between clinical parameters and DNA methylation levels in asthma patients.

|  |  | *p*-value | | | | | | | |
| --- | --- | --- | --- | --- | --- | --- | --- | --- | --- |
| CpG site | Gene | FEV1 | FEV1% | FEV1/FVC | PEF | FVC | FEF75 | FEF50 | FEF25 |
| **Chr4:75310649-1** | AREG | 0.309 | 0.105 | 0.093 | 0.33 | 0.933 | 0.025* | 0.019* | 0.030* |
| **Chr20:32274088** | E2F1 | 0.035* | 0.233 | 0.223 | 0.022* | 0.051 | 0.05 | 0.05 | 0.068 |
| **Chr20:32274358** | E2F1 | 0.113 | 0.059 | 0.968 | 0.182 | 0.033* | 0.306 | 0.543 | 0.641 |
| **Chr6:108883024** | FOXO3 | 0.044* | 0.032* | 0.063 | 0.038* | 0.238 | 0.758 | 0.195 | 0.05 |
| **Chr6:108882977** | FOXO3 | 0.063 | 0.011* | 0.055 | 0.048* | 0.366 | 0.949 | 0.147 | 0.051 |
| **Chr16:55514392** | MMP2 | 0.064 | 0.243 | 0.424 | 0.104 | 0.036* | 0.932 | 0.365 | 0.223 |
| **Chr16:55514437** | MMP2 | 0.151 | 0.198 | 0.75 | 0.102 | 0.025* | 0.343 | 0.489 | 0.246 |
| **Chr1:163291825** | NUF2 | 0.508 | 0.038* | 0.157 | 0.202 | 0.793 | 0.106 | 0.278 | 0.366 |
| **Chr17:7591672** | TP53 | 0.001* | 0.113 | 0.575 | 0.004* | 0.0001* | 0.758 | 0.171 | 0.019* |

**p*-value < 0.05 was considered statistically significant.
