# Supplementary material for: Aberrant Methylation of Aging-Related Genes in Asthma
Source: Front Mol Biosci. 2021 May 25;8:655285. doi: 10.3389/fmolb.2021.655285 (PMC8203316; doi:10.3389/fmolb.2021.655285)
Supplement: Supplementary file 6 [file Table4.DOCX]

**Table 4.** The top 9 differentially methylated sites of the differential aging-related genes associated with asthma.

| CpG site | Gene | AUC | *p*-value | Optimal diagnostic threshold | Sensitivity | Specificity |
| --- | --- | --- | --- | --- | --- | --- |
| **Chr4:75310649-1** | AREG | 0.716 | 0.009* | 0.086 | 0.724 | 0.81 |
| **Chr20:32274088** | E2F1 | 0.717 | 0.009* | 0.009 | 0.517 | 0.857 |
| **Chr20:32274358** | E2F1 | 0.746 | 0.022* | 0.043 | 1 | 0.533 |
| **Chr6:108883024** | FOXO3 | 0.653 | 0.066 | 0.166 | 0.909 | 0.667 |
| **Chr6:108882977** | FOXO3 | 0.671 | 0.040* | 0.263 | 0.69 | 0.714 |
| **Chr16:55514392** | MMP2 | 0.763 | 0.038* | 0.038 | 0.69 | 0.614 |
| **Chr16:55514437** | MMP2 | 0.688 | 0.024* | 0.017 | 0.414 | 1 |
| **Chr1:163291825** | NUF2 | 0.708 | 0.010* | 0.012 | 0.862 | 0.571 |
| **Chr17:7591672** | TP53 | 0.721 | 0.008* | 0.015 | 0.966 | 0.476 |

Statistics were done by spss 22.0, *p-*value < 0.05 was considered statistically significant.
